# Synergistic Regulation of Ionic Defects Enabled by NdCl_3_ for Efficient and Stable Wide-Bandgap Perovskite Solar Cells

**DOI:** 10.3390/ma19194184

**Published:** 2026-09-30

**Authors:** Qi Cui, Haoran Huang, Yu Chen, Xu Liu, Jincheng Huang, Jianlin Chen, Zhuoyin Peng

**Affiliations:** 1 School of Energy and Power Engineering, Changsha University of Science and Technology, Changsha 410111, China; 2 School of Civil and Environmental Engineering, Changsha University of Science and Technology, Changsha 410111, China

**Keywords:** perovskite solar cells, wide-bandgap perovskites, ionic-defect regulation

## Abstract

Wide-bandgap mixed-halide perovskites are attractive top-cell absorbers for tandem photovoltaics owing to their high-voltage potential and complementary spectral response. However, ionic defects generated during solution processing promote nonradiative recombination, ion migration, etc. Here, NdCl_3_ is introduced into Cs_0.17_FA_0.83_PbI_1.8_Br_1.2_ perovskites to synergistically regulate ionic defects. Structural analyses suggest that Nd^3+^ preferentially incorporates into interstitial sites, where its strong electrostatic interaction with neighboring halide ions may contribute to restricting their migration. The reduced defect density observed upon NdCl_3_ modification points to effective defect passivation as a primary outcome. Meanwhile, Cl^−^ compensates for halide vacancies, improves the local coordination environment, and suppresses trap-assisted nonradiative recombination. This synergistic regulation promotes grain growth, reduces defect density, facilitates carrier transport, and improves interfacial energy-level alignment. Consequently, the NdCl_3_-modified device achieves the highest power conversion efficiency of 19.87%. Moreover, the unencapsulated device retains 92.5% of its initial efficiency after 1680 h of ambient shelf storage (~25 °C, ~20% RH). These results demonstrate that the cooperative regulation of cation-associated ion migration and anion vacancies provides an effective route toward efficient and stable wide-bandgap perovskite photovoltaics.

## 1. Introduction

Photovoltaic technology has spawned diverse material-system generations. First-generation silicon-based cells have long dominated the market, with continuous optimization of silicon-wafer processing enabling cost reduction and efficiency enhancement [1]. Second-generation thin-film cells employ metal oxides (e.g., TiO_2_, ZnO, WO_3_) as functional layers, which serve key roles such as transparent electrodes and electron-transport layers [2]. Based on these two material systems, organic-inorganic hybrid perovskite solar cells have emerged as one of the most promising next-generation photovoltaic technologies owing to their excellent optoelectronic properties, tunable compositions, and compatibility with low-cost solution processing [3,4]. Within this family, wide-bandgap perovskites (typically >1.65 eV) have attracted particular interest as top-cell absorbers in tandem solar cells, because they can complement the spectral response of narrow-bandgap bottom cells (e.g., silicon or low-bandgap perovskites) and deliver high open-circuit voltages (V_OC_) essential for achieving efficiencies beyond the Shockley-Queisser limit [5,6]. Among them, mixed-cation/mixed-halide compositions such as Cs_1−x_FA_x_PbI_y_Br_3−y_ offer a favorable combination of bandgap tunability, phase stability, and solution processability and have therefore become representative absorber systems for wide-bandgap perovskite solar cells [7,8]. Despite these advantages, their photovoltaic performance remains limited by pronounced voltage losses and insufficient operational stability.

A major origin of these limitations is the high density and mobility of ionic defects inherent to multicomponent mixed-halide perovskites. During solution processing and subsequent crystallization, nonstoichiometric defects, especially halide vacancies, can readily form within the perovskite lattice [9,10]. These vacancies not only act as electronically active trap sites that promote trap-assisted nonradiative recombination, but also provide energetically favorable pathways for halide-ion migration [11,12,13]. Under illumination, thermal stress, or an external electric field, the migration and redistribution of halide ions can induce local compositional fluctuations and facilitate halide phase segregation, ultimately deteriorating both device efficiency and stability [14,15].

Additive engineering provides a versatile approach for manipulating defect chemistry in perovskite films [16,17,18]. Among various additive engineering strategies, rare-earth (RE) ion doping has attracted growing interest due to the unique electronic configurations and multifunctional roles of lanthanide ions (Ln^3+^) in perovskites [19]. Beyond conventional spectral up- and down-conversion functionalities, recent studies demonstrate that RE^3+^ ions can be co-doped with other metal ions (e.g., Sb^3+^) to modulate photoluminescence and energy-transfer processes, or incorporated into perovskite lattices to construct chiral optical structures featuring tunable circularly polarized luminescence [20,21]. Meanwhile, trivalent-cation additive approaches have recently emerged as an important strategy to mitigate defects and suppress ion migration in perovskite solar cells. Wang et al. employed the trivalent cation ytterbium (Yb^3+^) as a modifier for the post-treatment of inorganic perovskite surfaces to fabricate highly efficient and stable inorganic perovskite solar cells (PSCs) [22]. Zhao et al. verified that Nd^3+^ ions occupying the interstitial sites of the perovskite lattice can effectively suppress ion migration and greatly enhance device stability [23]. Chloride-containing additives have been extensively employed to regulate perovskite crystallization and reduce halide-related defects. Chloride species can modify the local coordination environment, promote high-quality film formation, and suppress defect-assisted recombination [24,25]. Nevertheless, most previous strategies predominantly focus either on restricting ion migration through cationic regulation or on improving crystallization and defect passivation through chloride additives. Although recent studies have reported multifunctional additives that simultaneously passivate defects and suppress ion migration in wide-bandgap perovskites, these strategies rely on distinct working mechanisms [26,27]. To date, no systematic demonstration has been established for the specific Cs_0.17_FA_0.83_PbI_1.8_Br_1.2_ composition via the synergistic effect of trivalent rare-earth cation interstitial occupation and chloride anion vacancy compensation. Considering the intrinsic coupling between halide vacancies and ionic migration, a single additive capable of simultaneously addressing both processes would provide a more effective route for stabilizing wide-bandgap mixed-halide perovskites.

Here, we introduce NdCl_3_ into Cs_0.17_FA_0.83_PbI_1.8_Br_1.2_ wide-bandgap perovskites as a dual-functional ionic-defect regulator. The Nd^3+^ and Cl^−^ species play complementary roles in regulating the defect chemistry of the perovskite lattice. Structural analyses suggest that Nd^3+^ preferentially occupies interstitial sites, where its high positive charge may strengthen the local electrostatic interaction with neighboring halide ions and potentially restrict their migration. Simultaneously, Cl^−^ compensates halide-vacancy defects and improves the local Pb-halide coordination environment, thereby suppressing trap-assisted nonradiative recombination. Such synergistic regulation promotes grain growth, reduces defect density, improves carrier transport, and optimizes interfacial energetics. Consequently, the NdCl_3_-modified device achieves the highest power conversion efficiency of 19.87%. Notably, after 1680 h of storage under ambient conditions, the unencapsulated devices retained over 92.5% of their initial efficiency, fully demonstrating the effectiveness of the NdCl_3_ modification strategy in improving device stability.

## 2. Experimental Section

### 2.1. Materials

Cesium iodide (CsI), lead iodide (PbI_2_), lead bromide (PbBr_2_), methylammonium chloride (MACl), and formamidinium iodide (FAI) were purchased from Liaoning Youxuan New Energy Technology Co., Ltd. (Yingkou, China). Neodymium (III) chloride (NdCl_3_), N,N-dimethylformamide (DMF), dimethyl sulfoxide (DMSO), isopropanol (IPA), ethyl acetate, bathocuproine (BCP), and [6,6]-phenyl-C_61_-butyric acid methyl ester (PCBM) were purchased from Sigma-Aldrich (St. Louis, MO, USA). Poly[bis(4-phenyl)(2,4,6-trimethylphenyl)amine] (PTAA) was purchased from Luminescence Technology Corp. (New Taipei City, Taiwan, China).

### 2.2. Device Fabrication

The ITO substrates were cleaned by sequential ultrasonication in deionized water, detergent, and isopropanol (30 min each), and then dried under a nitrogen flow. Prior to use, the cleaned ITO substrates were treated with UV-ozone for 25 min. A chlorobenzene solution of PTAA (1.5 mg mL^−1^) was deposited onto the ITO substrates by a two-step spin-coating process (1000 rpm, 3 s; 6000 rpm, 30 s), and the films were annealed at 100 °C for 15 min to form the hole-transport layer. The perovskite precursor solution was prepared by dissolving CsI, FAI, PbI_2_, and PbBr_2_ in a mixed solvent of DMF and DMSO (9:1, volume ratio). For NdCl_3_-modified films, NdCl_3_ was added to the precursor solution at a concentration of 0.5 mg mL^−1^. The perovskite layer (Cs_0.17_FA_0.83_PbI_1.8_Br_1.2_) was deposited using an antisolvent-assisted crystallization process. The as-prepared hybrid perovskite precursor solution was spin-coated onto the PTAA/ITO substrates at 4000 rpm for 30 s, and 100 μL of ethyl acetate was dripped as the antisolvent 10 s before the end of the spin-coating process to promote rapid nucleation and crystallization. The as-cast films were subsequently annealed at 100 °C for 20 min, yielding uniform and compact perovskite layers. Subsequently, a PCBM solution in chlorobenzene (20 mg mL^−1^) was spin-coated onto the perovskite layer at 2000 rpm for 40 s, followed by annealing at 80 °C for 5 min. A BCP solution in isopropanol (0.5 mg mL^−1^) was then spin-coated onto the PCBM layer at 4000 rpm for 30 s without further annealing. Finally, a 100 nm Ag electrode was thermally evaporated under a vacuum below 10^−4^ Pa to complete device fabrication.

### 2.3. Characterization

UPS measurements were carried out on an AXIS Supra system equipped with a dedicated accessory. XPS analysis was performed using a Thermo Scientific K-Alpha spectrometer (Thermo Scientific, East Grinstead, UK). XPS data were processed using Avantage software (v5.9931). All spectra were calibrated by setting the adventitious C 1s peak to 284.8 eV. Background subtraction was performed using a Shirley-type background. Peak fitting was carried out using mixed Gaussian-Lorentzian (GL) line shapes with fixed area ratios for spin–orbit splitting doublets. Full XPS survey spectra are provided in Figure S1. The spin–orbit splitting energies (Δ) for each core level were extracted from the fitted spectra and are summarized in Table S1. UV-vis absorption spectra were obtained with a Shimadzu UV-3600 Plus spectrophotometer (Tokyo, Japan). PL and TRPL measurements were conducted using an F-7000 fluorescence spectrophotometer (Livingston, UK). XRD patterns were collected with a DX-2700BH diffractometer (Dandong, China). KPFM measurements were performed on a Bruker Dimension Icon system (Karlsruhe, Germany). SEM images were acquired using a MIRA4 LMH microscope (Brno, Czech Republic) at an accelerating voltage of 5 kV. EQE spectra were recorded with a SCS600 system (Zolix Instruments Co., Ltd., Beijing, China). EIS measurements were conducted in the dark using PP211 and CHI1030B electrochemical workstations (Zahner Electrochemistry, Kronach, Germany). The spectra were recorded over a frequency range of 100 mHz to 100 kHz with an AC perturbation amplitude of 10 mV under open-circuit conditions. The obtained spectra were fitted using the equivalent circuit model R(CR)(CR), and the fitting results are summarized in Table S2. Mott-Schottky measurements were performed using an AC amplitude of 30 mV at a frequency of 10 kHz. J-V curves and dark current were measured under AM 1.5G illumination (100 mW cm^−2^) using a Keithley 2400 solar cell test system (Beijing Zhongguo Jinyuan Technology Co., Ltd., Beijing, China).

## 3. Result and Discussion

**Figure 1:**
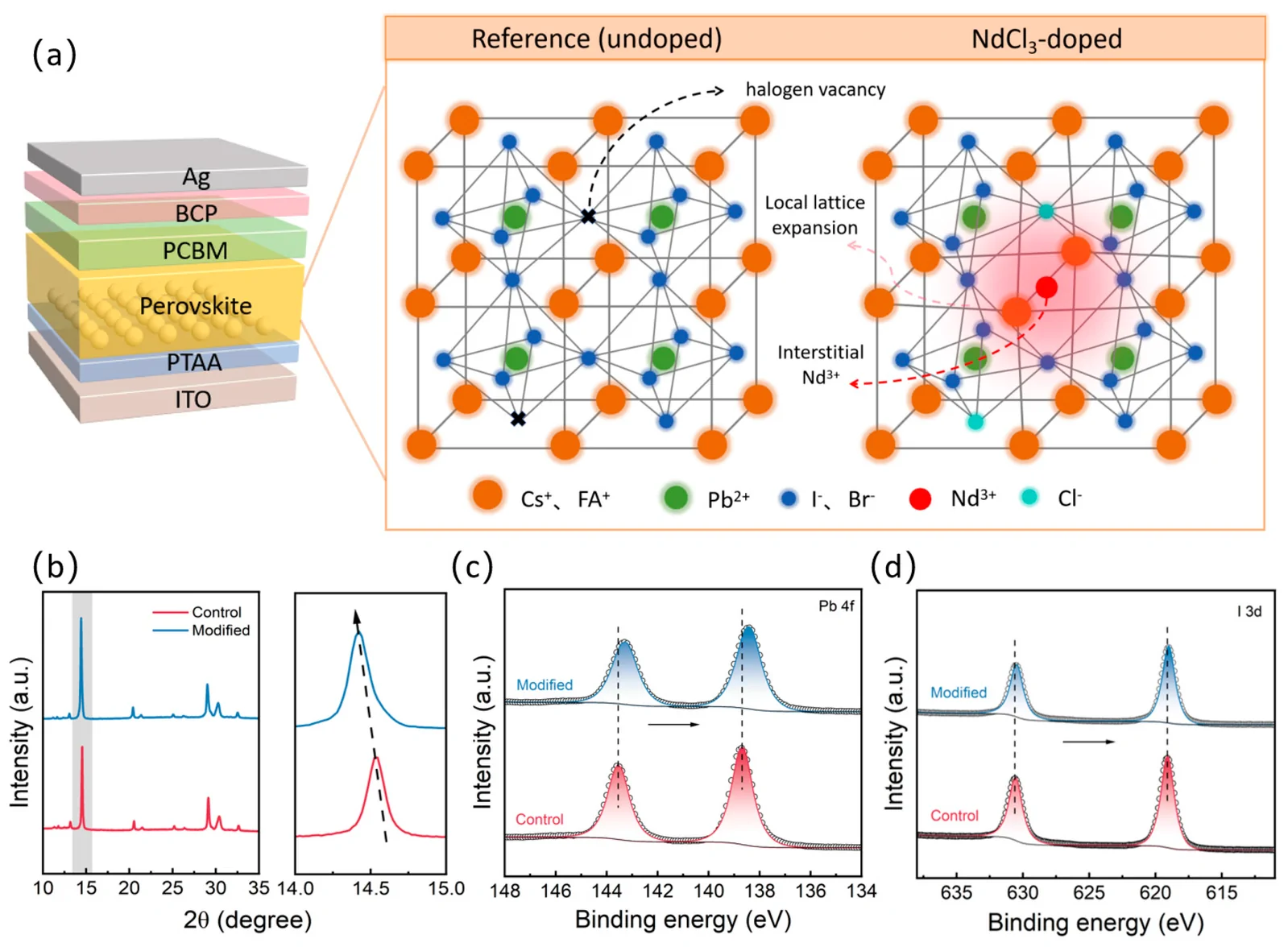
(**a**) Schematic illustration of the device architecture and proposed NdCl_3_-assisted ionic-defect regulation mechanism. (**b**) The XRD patterns. The XPS spectra of (**c**) Pb 4f and (**d**) I 3d.

Figure 1a schematically illustrates the inverted device architecture employed in this work, ITO/PTAA/perovskite (w or w/o NdCl_3_)/PCBM/BCP/Ag, together with the proposed mechanism of NdCl_3_-assisted ionic-defect regulation. In the control perovskite, halide-vacancy defects act both as electronic trap states and favorable pathways for halide-ion migration. Upon incorporation of NdCl_3_, the additive dissociates into Nd^3+^ and Cl^−^ species that play complementary roles. Cl^−^ compensates halide vacancies and improves the local coordination environment, whereas Nd^3+^ is proposed to preferentially occupy interstitial sites and interact electrostatically with neighboring halide ions, thereby inducing local lattice expansion, modulating the intrinsic microstrain and restricting their migration.

To clarify the proposed interstitial incorporation of Nd^3+^ in the Cs_0.17_FA_0.83_PbI_1.8_Br_1.2_ lattice, we analyzed the lattice from the perspectives of ionic-size compatibility, tolerance-factor variation, and XRD characteristics. The effective ionic radii of Nd^3+^ and Pb^2+^ are 98.3 pm and 119 pm [28,29]. For hypothetical substitution of Pb^2+^ by Nd^3+^ at the B-site, the calculated tolerance factor is approximately 1.028, exceeding the empirical stability range generally associated with the three-dimensional perovskite framework [30]. In addition, the substantial size and valence mismatch between Nd^3+^ and Pb^2+^ makes direct B-site substitution unfavorable. Consistent with this interpretation, the (100) diffraction peak of the NdCl_3_-modified film shifts toward lower 2θ angles (Figure 1b), indicating a slight lattice expansion rather than the lattice contraction expected for substitution by the smaller Nd^3+^ ion. These results collectively support preferential interstitial incorporation of Nd^3+^ in the perovskite lattice.

The influence of NdCl_3_ on the local chemical environment was further examined by XPS. Figure 1c,d and Figure S2 show the high-resolution XPS spectra of Pb 4f, I 3d, and Br 3d, respectively. Following NdCl_3_ modification, all characteristic peaks shift toward lower binding energies, indicating a redistribution of the local electronic environment within the Pb–halide framework and altered interactions between the introduced species and the perovskite lattice. These changes suggest improved local coordination and reduced electronic deficiency and are consistent with a possible vacancy-compensation mechanism. Together with the structural and electrical results, these observations are consistent with the proposed cooperative effects of Nd^3+^ incorporation and Cl^−^-mediated regulation of halide-related defects. To further verify the incorporation of Nd and Cl into the perovskite bulk, XPS depth-profiling measurements were performed on the NdCl_3_-modified films. As shown in Figures S3 and S4, both Nd 3d and Cl 2p signals are detected at sputtering depths of 0 s, 30 s, and 60 s, confirming that Nd^3+^ and Cl^−^ are distributed throughout the bulk of the perovskite film rather than being confined to the surface. To examine the spatial distribution of Nd and Cl within the perovskite film, SEM-EDS was performed on the NdCl_3_-modified sample. As shown in Figures S5 and S6, both Nd and Cl exhibit relatively uniform distributions across the examined region, without detectable microscale enrichment at grain boundaries, interfaces, or other localized regions. Moreover, XPS depth profiling reveals persistent Nd and Cl signals after progressive sputtering, confirming their presence within the film rather than exclusive localization at the surface.

**Figure 2:**
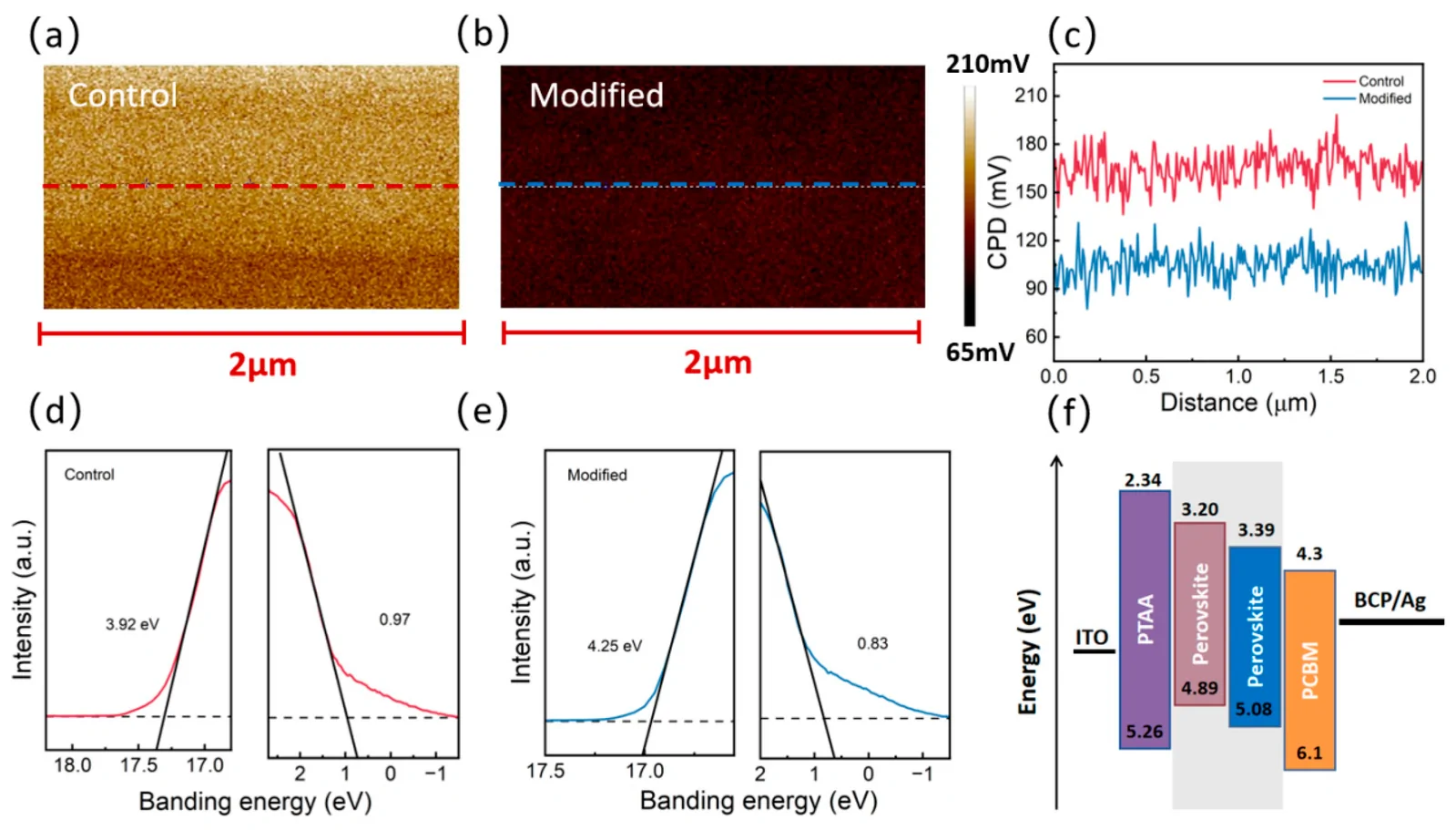
KPFM images of (**a**) control and (**b**) modified perovskite films. (**c**) Corresponding CPD distributions. UPS spectra of (**d**) control and (**e**) modified perovskites. (**f**) Energy level diagrams.

To determine how NdCl_3_-induced defect regulation affects the surface electronic structure and interfacial energetics, KPFM and UPS measurements were performed. Figure 2a–c show that the contact potential difference (CPD) of the modified film decreases relative to that of the control film. Under the same measurement configuration, this variation corresponds to an increase in the surface work function, suggesting a downward shift of the surface Fermi level after NdCl_3_ modification. UPS measurements further substantiate this change. As shown in Figure 2d,e, the work function increases from 3.92 eV to 4.25 eV after NdCl_3_ incorporation. Meanwhile, the energy gap between the valence-band maximum (VBM) and Fermi level (E_F_) decreases from 0.97 eV to 0.83 eV, indicating a shift of E_F_ toward the VBM. Because the optical bandgap remains essentially unchanged, these results indicate an overall downward shift of the perovskite energy levels (Figure 2f).

This energetic modulation is favorable for charge extraction at both charge-selective interfaces, as it reduces the energetic offsets for electron transfer to PCBM and hole transfer to PTAA. At the perovskite/PCBM interface, the conduction-band minimum (CBM) shifts from −3.20 eV to −3.39 eV, reducing the energetic offset relative to the PCBM LUMO (−4.30 eV) from 1.10 to 0.91 eV. This reduced offset is expected to be favorable for electron transfer from the perovskite into PCBM, although the actual charge-transfer efficiency also depends on interfacial chemistry and band-bending effects. At the PTAA/perovskite interface, the VBM shifts from −4.89 eV to −5.08 eV, decreasing the energetic offset relative to the PTAA HOMO (−5.26 eV) from 0.37 eV to 0.18 eV. The improved energetic matching is therefore favorable for hole extraction and for reducing charge accumulation and recombination at the buried interface. We note, however, that the above discussion is based on static energy offsets derived from UPS measurements, which do not fully capture the actual charge-transfer dynamics at the interfaces. Factors such as interface dipoles, band bending, and interfacial defect states can significantly influence carrier extraction behavior. The observed work-function change upon NdCl_3_ modification is consistent with improved energetic alignment, but a more complete understanding of the interfacial charge-transfer processes would require further investigation. Nevertheless, the observed work-function change and reduced energetic offsets upon NdCl_3_ modification are consistent with more favorable interfacial energetics, which may contribute to improved charge extraction.

The increase in work function observed by UPS is also consistent with the KPFM results and with the XPS-detected modification of the local electronic environment. Halide vacancies can exhibit donor-like behavior and perturb the Fermi-level position. Their compensation by Cl^−^ reduces defect-induced electronic doping and restores a more favorable surface electronic structure. NdCl_3_ modification therefore affects not only the local chemical coordination of the perovskite but also its macroscopic surface energetics, providing a favorable basis for improved carrier extraction.

**Figure 3:**
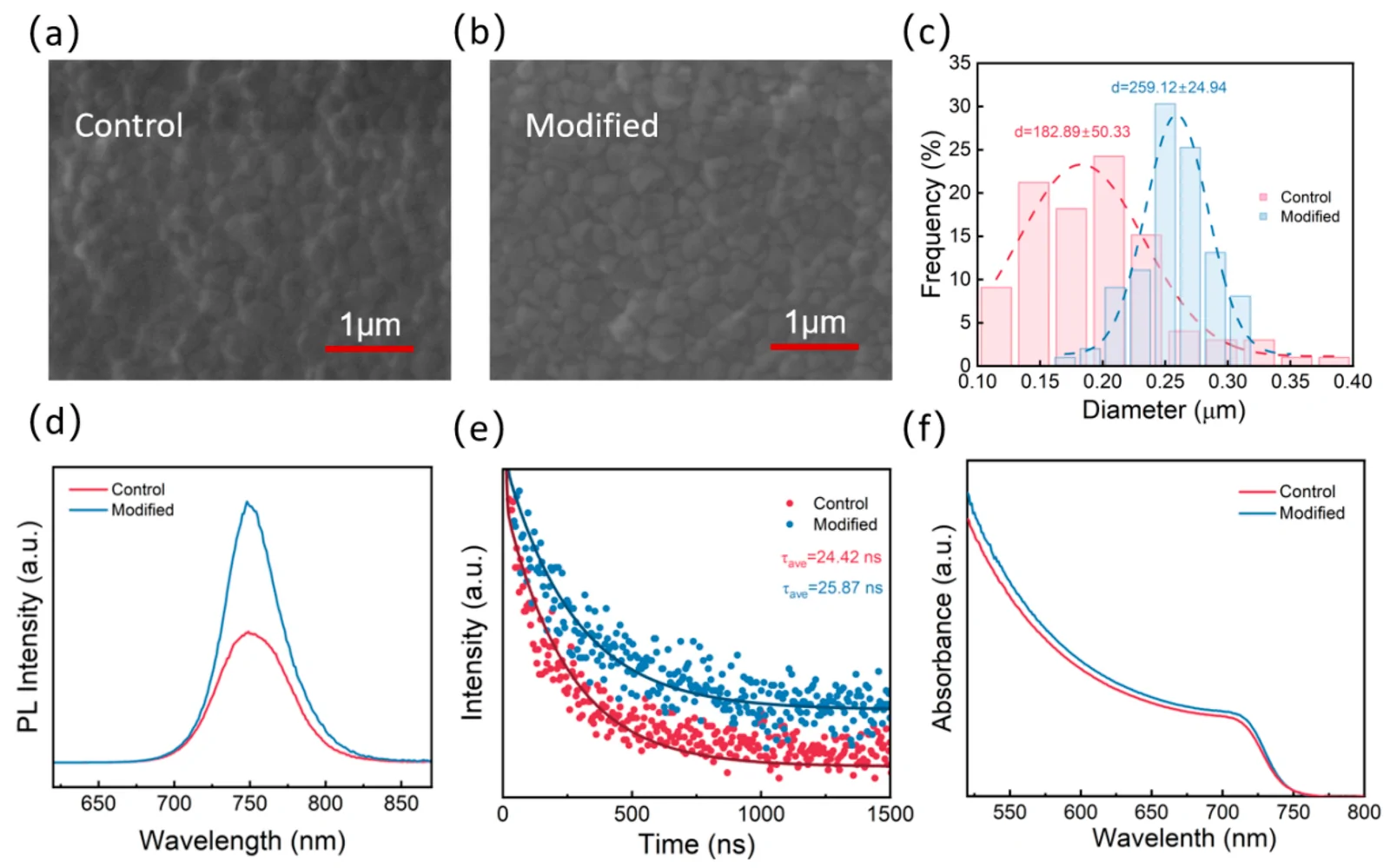
Top-view SEM images of (**a**) control and (**b**) modified perovskite films. (**c**) Corresponding grain-size distributions. (**d**) Steady-state PL spectra. (**e**) TRPL decay curves. (**f**) UV-vis absorption spectra.

The influence of NdCl_3_ on perovskite crystallization and film morphology was subsequently investigated. Top-view SEM images (Figure 3a,b) reveal a pronounced increase in grain size after NdCl_3_ modification. The modified film exhibits larger and more uniformly distributed grains with fewer grain boundaries and no apparent surface pinholes. Statistical analysis (Figure 3c) shows that the average grain size increases from 182.89 nm to 259.12 nm. Such grain enlargement decreases the density of grain-boundary regions, which commonly host structural defects and facilitate ion migration. Cross-sectional SEM images (Figure S7) further demonstrate that both films possess dense columnar morphologies. Nevertheless, localized interfacial discontinuities are visible in the control film, whereas the NdCl_3_-modified film exhibits more intimate contact with the adjacent PTAA and PCBM/BCP layers, with no obvious interfacial voids or delamination. In addition, more continuous grains extend across the perovskite thickness after modification. This vertically connected morphology is favorable for carrier transport and reduces the probability of grain-boundary-mediated recombination.

The optoelectronic quality of the films was evaluated using steady-state PL and TRPL measurements. As shown in Figure 3d, the NdCl_3_-modified film exhibits enhanced PL intensity without an appreciable shift in the emission peak, indicating that NdCl_3_ suppresses nonradiative recombination without significantly altering the intrinsic bandgap. The TRPL curves in Figure 3e provide further support for this interpretation. The average carrier lifetime increases modestly from 24.42 ns to 25.87 ns after NdCl_3_ modification. This moderate but reproducible enhancement is consistent with the suppression of nonradiative recombination, although the magnitude of the change is relatively small. The UV-vis absorption spectra (Figure 3f) show nearly identical absorption edges and comparable visible-light absorption for the two films. Therefore, trace NdCl_3_ modification improves film morphology and electronic quality while preserving the intrinsic optical absorption characteristics of the wide-bandgap perovskite.

**Figure 4:**
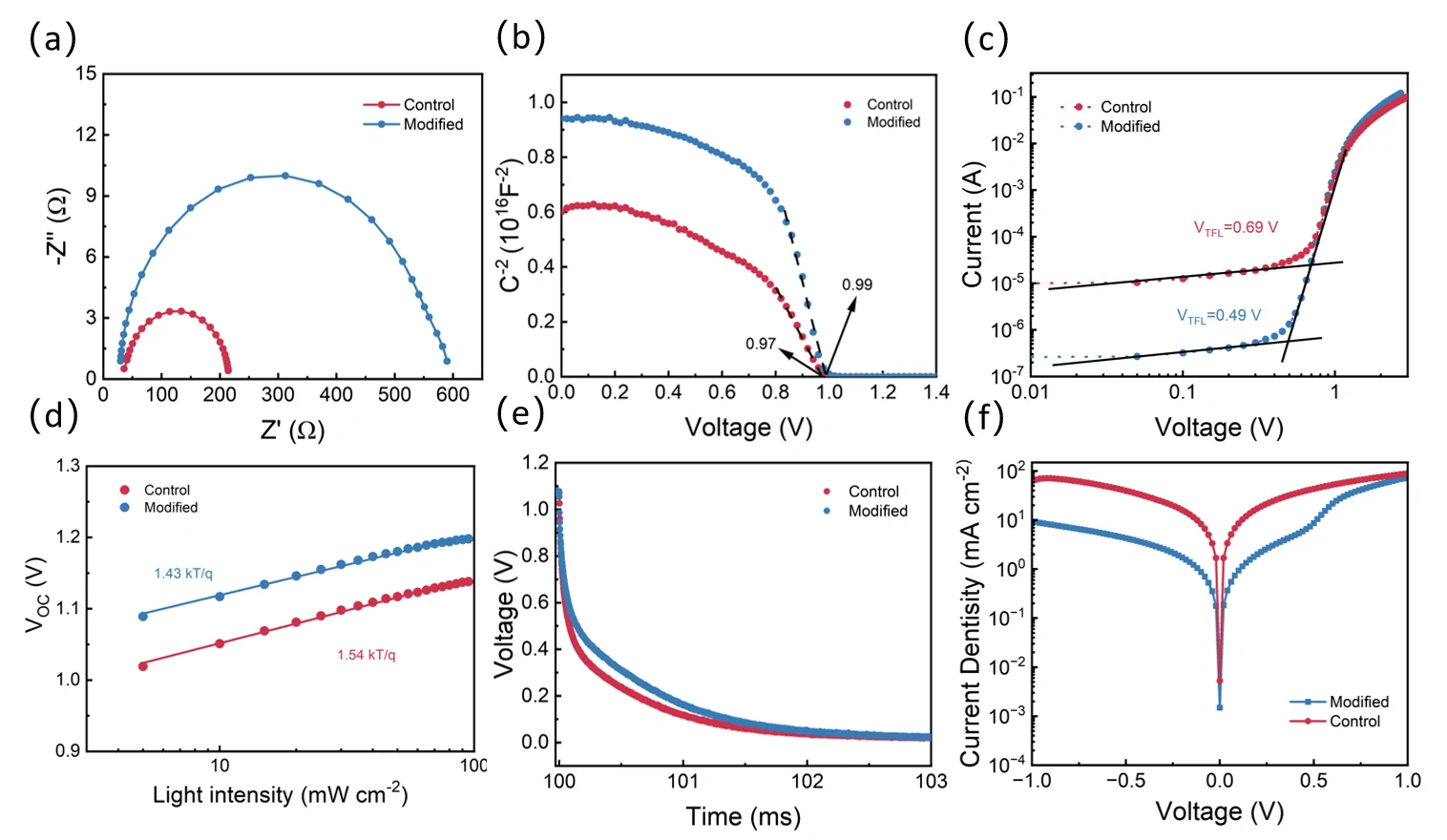
(**a**) EIS spectra. (**b**) Mott-Schottky plots. (**c**) SCLC curves. (**d**) V_OC_ as a function of light intensity. (**e**) TPV spectra. (**f**) Dark J-V curves.

The effect of NdCl_3_-induced defect regulation on charge-carrier dynamics was investigated. Figure 4a shows the EIS spectra of the corresponding devices. The NdCl_3_-modified device exhibits a larger recombination resistance than the control device, indicating suppressed charge recombination. This behavior is consistent with the reduction in electronically active defects following NdCl_3_ modification. The Mott-Schottky plots in Figure 4b provide further insight into the internal electric field. The built-in potential ($V_{\mathrm{bi}}$) extracted from the Mott-Schottky plots increases slightly from 0.97 V to 0.99 V after modification. This modest change suggests a minor improvement in the internal electric field, which may be favorable for carrier separation and extraction. Moreover, the increased slope of the Mott-Schottky plot after modification suggests a lower background carrier concentration, consistent with the suppression of defect-induced electronic doping associated with halide vacancies.

The bulk trap density was quantitatively evaluated using space-charge-limited current (SCLC) measurements in hole-only devices (Figure 4c). The trap-filled limit voltage ($V_{\mathrm{TFL}}$) was used to quantitatively evaluate the trap-state density within the perovskite film, which was calculated using the following formula [31]:

(1) $$N_{\mathrm{trap}}=\frac{2V_{\mathrm{TFL}}\varepsilon \varepsilon_{0}}{qL^{2}}$$

$N_{\mathrm{trap}}$ is the trap-state density, L is the perovskite film thickness, q is the elementary charge, ε and $\varepsilon_{0}$ are the relative permittivity of the perovskite material and the vacuum permittivity, respectively, and $V_{\mathrm{TFL}}$ is the trap-filled limit voltage. Here, $\varepsilon_{0}$ = 8.854 × 10^−14^ F cm^−1^, ε = 8.0, q = 1.602 × 10^−19^ C, and *L* = 500 nm. According to the formula, the $V_{\mathrm{TFL}}$ of the control device is 0.69 V, which decreases to 0.49 V after NdCl_3_ modification. Using these parameters, the calculated trap densities are 2.44 × 10^17^ cm^−3^ for the control device and 1.73 × 10^17^ cm^−3^ for the NdCl_3_-modified device. The lower $V_{\mathrm{TFL}}$ observed in the modified sample directly demonstrates a significant reduction in the bulk defect concentration within the perovskite film. This conclusion is further supported by the lateral dark-conductivity measurements of ITO/perovskite/Ag devices (Figure S8), for which the NdCl_3_-modified film exhibits a steeper current-voltage response. The enhanced conductivity suggests more efficient carrier transport as a consequence of reduced trap-mediated carrier localization.

Light-intensity-dependent measurements were further employed to analyze recombination behavior (Figure 4d). The ideality factor (n) decreases from 1.54 for the control device to 1.43 for the NdCl_3_-modified device, indicating reduced trap-assisted recombination. Consistent with this result, transient photovoltage (TPV) and transient photocurrent (TPC) measurements (Figure 4e and Figure S9) show that the modified device exhibits a longer recombination lifetime together with faster charge extraction. These observations demonstrate that NdCl_3_ simultaneously suppresses carrier recombination and facilitates charge transport. The dark J-V characteristics provide additional evidence for defect suppression (Figure 4f). Under reverse bias, the NdCl_3_-modified device exhibits a substantially lower leakage current than the control device, indicating suppression of defect-mediated leakage pathways and trap-assisted tunneling.

**Figure 5:**
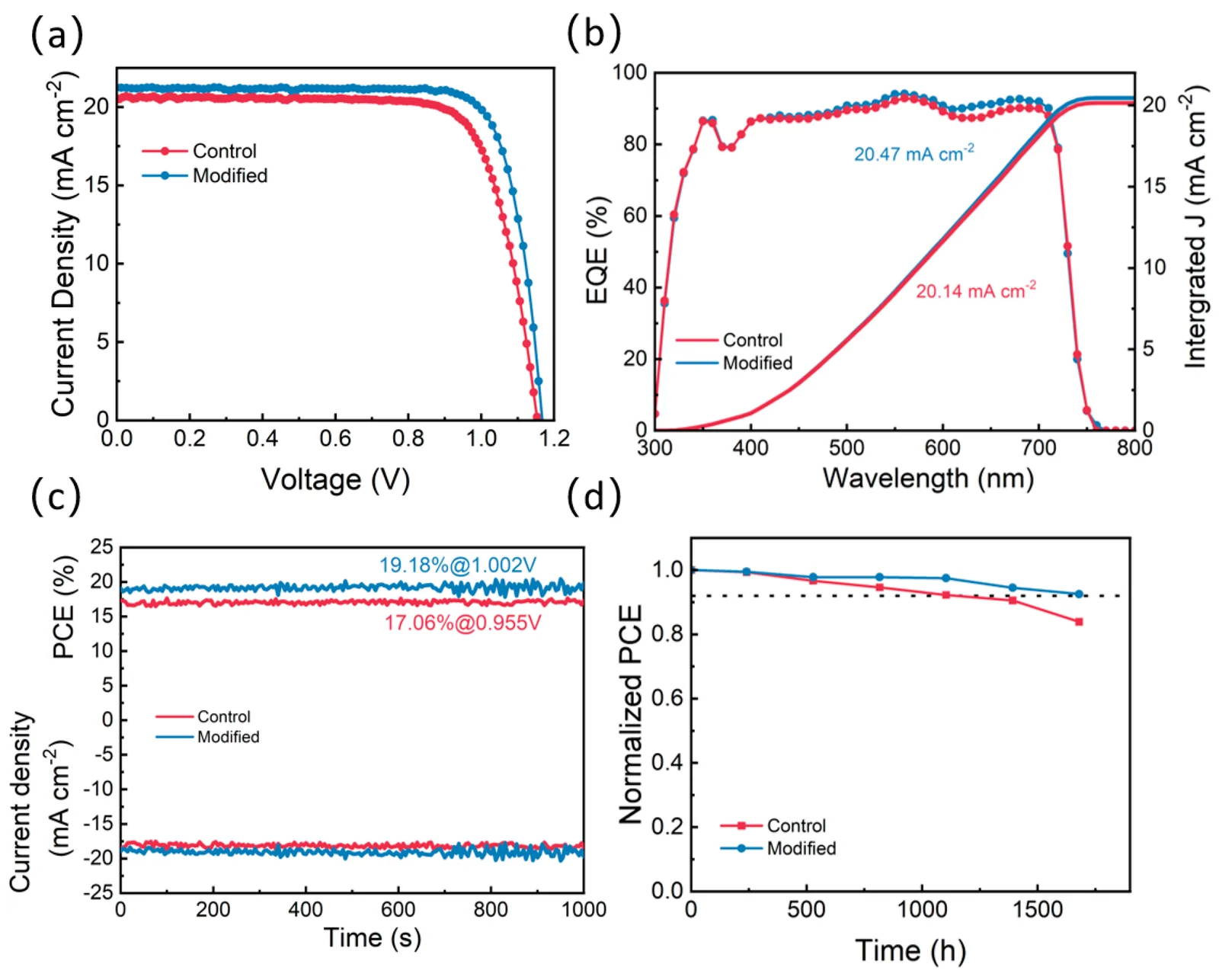
(**a**) J-V curves of the best-performing devices. (**b**) EQE spectra and integrated current densities. (**c**) Stabilized power output at the maximum power point. (**d**) Long-term stability of the unencapsulated devices.

The improvements in film quality, defect chemistry, and carrier dynamics are directly reflected in device performance. Figure 5a presents the J-V characteristics of the best-performing devices. The control device exhibits a maximum power conversion efficiency (PCE) of 18.19%, with a short-circuit current density (J_SC_) of 20.57 mA cm^−2^, an open-circuit voltage (V_OC_) of 1.153 V, and a fill factor (FF) of 76.69%. After NdCl_3_ modification, the highest PCE increases to 19.87%, accompanied by a J_SC_ of 20.91 mA cm^−2^, a V_OC_ of 1.172 V, and an FF of 81.12%. The EQE spectra in Figure 5b show an enhanced spectral response for the modified device across most of the 300–780 nm region. The integrated J_SC_ values are 20.14 mA cm^−2^ and 20.47 mA cm^−2^ for the control and modified devices, respectively, in good agreement with the corresponding J-V measurements. To evaluate device reproducibility, 14 control devices and 14 NdCl_3_-modified devices were fabricated, and their photovoltaic parameter distributions are summarized in Figure S10. The control devices exhibit an average PCE of 17.05 ± 0.92%, while the modified devices deliver an average PCE of 19.42 ± 0.46%.

The forward and reverse J-V scan results shown in Figure S11 reveal reduced hysteresis after NdCl_3_ incorporation, which is often associated with mitigated ion migration. However, we note that reduced hysteresis alone does not directly confirm suppressed ion mobility; it may also reflect improved charge extraction and reduced interfacial charge accumulation resulting from the lower defect density. Stabilized power output (SPO) measurements were conducted at the maximum power point to assess short-term operational behavior (Figure 5c). Under continuous illumination for 1000 s, the control device maintains a stabilized PCE of 17.06% at a bias voltage of 0.955 V, whereas the NdCl_3_-modified device delivers a stabilized efficiency of 19.18% at 1.002 V. The modified device maintains a consistently higher photocurrent and power output throughout the 1000 s measurement, confirming that the improvement observed from J-V scans translates into stable power output under sustained bias. The ambient storage stability (~25 °C, ~20% RH) of the unencapsulated devices is shown in Figure 5d. After 1680 h of storage under ambient conditions, the control device retains less than 84.0% of its initial efficiency, whereas the NdCl_3_-modified device retains 92.5%. The markedly improved stability is consistent with the proposed synergistic mechanism: compensation of halide vacancies reduces defect-assisted degradation and nonradiative recombination. The possible restriction of halide-ion migration by interstitial Nd^3+^, while not directly measured, may further contribute to mitigating composition redistribution. These results demonstrate that NdCl_3_-mediated ionic-defect regulation improves not only the initial photovoltaic performance but also the long-term stability of wide-bandgap mixed-halide perovskite solar cells.

## 4. Conclusions

In summary, we demonstrate a synergistic ionic-defect-regulation strategy for Cs_0.17_FA_0.83_PbI_1.8_Br_1.2_ wide-bandgap perovskite solar cells using NdCl_3_ as a dual-functional additive. Structural analyses suggest preferential incorporation of Nd^3+^ at interstitial sites within the perovskite lattice, where its strong electrostatic interaction with neighboring halide ions may contribute to hindering ionic migration. Simultaneously, Cl^−^ compensates halide-vacancy defects and improves the local Pb-halide coordination environment, thereby reducing trap-assisted nonradiative recombination. The cooperative regulation of these two ionic species promotes grain growth, lowers defect density, improves carrier transport, and optimizes interfacial energy-level alignment. Benefiting from these improvements, the optimized device achieves a maximum PCE of 19.87%. Moreover, the unencapsulated device retains 92.5% of its initial efficiency after 1680 h of ambient storage (~25 °C, ~20% RH). These results highlight the importance of simultaneously regulating defect concentration and ionic mobility and provide a rational design strategy for improving the efficiency and stability of wide-bandgap mixed-halide perovskite photovoltaics.

## Data Availability

The original contributions presented in this study are included in the article/Supplementary Material. Further inquiries can be directed to the corresponding authors.

## Supplementary Materials

The following supporting information can be downloaded at: https://www.mdpi.com/article/10.3390/ma19194184/s1, Figure S1. Full XPS survey spectra of control and NdCl3-modified perovskite. Figure S2. The XPS spectra of Br 3d. Figure S3. The XPS depth profiling spectra of Nd 3d. Figure S4. The XPS depth profiling spectra of Cl 2p. Figure S5. SEM-EDS elemental mapping of Nd. Figure S6. SEM-EDS elemental mapping of Cl. Figure S7. Cross-sectional SEM images. Figure S8. Dark current-voltage (I-V) curves. Figure S9. TPC spectra. Figure S10. Box plots of V_OC_, J_SC_, FF, and PCE for the control device and the modified device. Figure S11. Forward and reverse scan J-V curves. Table S1. XPS spin-orbit splitting energies (Δ) for control and NdCl_3_-modified perovskite films. Table S2. EIS fitting parameters for control and NdCl_3_-modified devices.

## References

[1] Cheng D., Gao Y., Yang C. (2025). A comprehensive review on wafering of silicon substrate for photovoltaic solar cell. Sol. Energy.

[2] Suchikova Y., Nazarovets S., Konuhova M., Popov A.I. (2025). Binary Oxide Ceramics (TiO_2_, ZnO, Al_2_O_3_, SiO_2_, CeO_2_, Fe_2_O_3_, and WO_3_) for Solar Cell Applications: A Comparative and Bibliometric Analysis. Ceramics.

[3] Liang L., Nan Z.-A., Li Y., Zhang Y., Fei Z., Shibayama N., Zhang Z., Lin Z., Chen W., Li C. (2025). Formation Dynamics of Thermally Stable 1D/3D Perovskite Interfaces for High-Performance Photovoltaics. Adv. Mater..

[4] Jiang X., Zeng J., Sun K., Li Z., Pan G., Guo R., Schwartzkopf M., Roth S.V., Xu B., Müller-Buschbaum P. (2025). Homogeneous FACsPbI3 Films via Sequential Deposition for Efficient and Stable Perovskite Solar Cells. Adv. Sci..

[5] Du H., Li J., Ma Z., Zhang Q., Gou F., Li Y., Chen B., Lv Z., Xiang D., Hou S. (2025). Suppressing Halide Segregation Via Dual-Anchoring Strategy for 31.20% Perovskite/Silicon Tandem Solar Cells. Adv. Energy Mater..

[6] Bi L., Wang J., Fu Q., Jen A.K.-Y. (2026). Crystallization Modulation for Stable Wide-Bandgap Perovskite Solar Cells and Modules. Adv. Mater..

[7] Zhao K., Yao L., Değer C., Zhang X., Shen J., Miao X., Shi P., Luo Y., Jin D., Tian Y. (2025). Mitigating residual MA+ for stable FAPbI3 perovskite photovoltaics. Nat. Commun..

[8] Ahmed Y., Qi X., Moazzezi P., Saidaminov M.I. (2026). Perovskite Photovoltaics: Pick FAPbI_3_ and Stick to It. ACS Energy Lett..

[9] Lu T., Dang Z., Luo Y., Li Y., Rao X., Zhang Z., Hu Y., Chen Z., Li Z., Petralanda U. (2025). Lattice expansion of hybrid perovskite inhibits halogen interstitial generation and enhances solar cell performance. Nat. Commun..

[10] Tan Y., Tian T., Chen H., Yang G., Yang M., Zhong J.-X., Chen Z.-R., He Y., Wu W.-Q. (2026). Supramolecular Chloride Reservoirs Enable Homogeneous Halide Distribution and Near-Unity Blue Luminescence in Cs_4_PbBr_6_-CsPbBr_3_ Heterostructured Perovskites. Angew. Chem. Int. Ed..

[11] Li Y., Sun N., Feng B., Li W., Cui Z., Zhang W., Fu S., Li X., Fang J. (2025). Wide-bandgap perovskite solar cells with > 1.34 V *V*_oc_ for all-perovskite tandems. Chem. Eng. J..

[12] Pan T., Zhou W., Wei Q., Peng Z., Wang H., Jiang X., Zang Z., Li H., Yu D., Zhou Q. (2023). Surface-Energy-Regulated Growth of α-Phase Cs_0.03_FA_0.97_PbI_3_ for Highly Efficient and Stable Inverted Perovskite Solar Cells. Adv. Mater..

[13] He D., Ma D., Zhang J., Yang Y., Ding J., Liu C., Liu X., Yu Y., Liu T., Chen C. (2025). Universal Ion Migration Suppression Strategy Based on Supramolecular Host–Guest Interaction for High-Performance Perovskite Solar Cells. Adv. Mater..

[14] Yuan B., Wang Z., Zhang S., Hofer C., Gao C., Chennit T., Shi H., Wu X., Han Y., Dou L. (2025). Atomically resolved edges and defects in lead halide perovskites. Nature.

[15] Zhang Z., Li M., Li R., Zhuang X., Wang C., Shang X., He D., Chen J., Chen C. (2024). Suppressing Ion Migration by Synergistic Engineering of Anion and Cation toward High-Performance Inverted Perovskite Solar Cells and Modules. Adv. Mater..

[16] Jiao B., Tan L., Ye Y., Ren N., Li M., Li H., Li X., Yi C. (2025). One-stone-two-birds: Over 26% efficiency in perovskite solar cells via synergistic crystallization & interface regulation. Energy Environ. Sci..

[17] Zhang F., Chen B., Chen J., Rao Y., Luo J., Guo R., Wang L. (2026). Crystallization regulation and defect passivation toward thermally evaporated pure-blue perovskite light-emitting diodes. Chem. Eng. J..

[18] Jiang Y., Zai H., Zheng X., Zhang Y., Faheem M.B., Dai S., Lv Z., Ma Y., Li L., Tao Y. (2026). Fused-Ring Electron Acceptors as a Versatile Additive Platform for Efficient Perovskite Photovoltaics. J. Am. Chem. Soc..

[19] An J., Pan G., Song H., Chen C. (2026). Lanthanide Ion-Doped Halide Perovskites and Their Optoelectronic Applications. Laser Photonics Rev..

[20] Wang H., Yao J., Yin Z., Cao S., Zhao J., Han X., Yu W.W., Zeng R. (2025). Symmetry Disruption and Charge Localization: Unraveling Roles of Sb^3+^ in Enhancing Luminescence of Rare Earth Double Perovskites. Laser Photonics Rev..

[21] Liang D., Peng F., Du Q., Zhao Y., Ma W. (2025). Gadolinium and Europium Doped Chiral Perovskite Films with Magnetic Circular Dichroism and Circularly Polarized Luminescence for Information Encryption Applications. Angew. Chem. Int. Ed..

[22] Wang Z., Xu T., Li N., Liu Y., Li K., Fan Z., Tan J., Chen D., Liu S., Xiang W. (2024). Interfacial engineering with trivalent cations for efficient and stable inverted inorganic perovskite solar cells. Energy Environ. Sci..

[23] Zhao Y., Yavuz I., Wang M., Weber M.H., Xu M., Lee J.-H., Tan S., Huang T., Meng D., Wang R. (2022). Suppressing ion migration in metal halide perovskite via interstitial doping with a trace amount of multivalent cations. Nat. Mater..

[24] Wang J., Hu S., Gu X., Truong M.A., Yang Y., Liu C., Kusch G., Yuan Z., Kober-Czerny M., Zhang Z. (2026). Homogenized Optoelectronic Properties in Perovskites: Achieving High-Efficiency Solar Cells with Common Chloride Additives. J. Am. Chem. Soc..

[25] Guo X., Jia Z., Dong Z., Kalasariya N., Hu J., Shi Z., Steele J.A., Chen Y.-H., Ochsner G., Chen J. (2026). Stable perovskite–organic tandem solar cells enabled by chloride-doped evaporated wide-bandgap perovskites. Energy Environ. Sci..

[26] Wu R., Qin S., Zou T., Jiang X., Wang S., Zhuang S., Li H., Wang Y., Li S., Liu M. (2026). Perovskite–organic tandem solar cells with a photo-transformable stabilizer. Nature.

[27] Song Y., Liu Z., Cai X., Ge H., Liu X., Wang X., Li A., Miyasaka T., Shibayama N., Wang X.-F. (2025). Efficient and Moisture Resistant Wide-Bandgap Perovskite Solar Cells with Phosphinate-Based Iodine Defect Passivation. Adv. Energy Mater..

[28] Liu D., Zhang S., Jing L., Wang H., Chen G., Xu L., Song H., Kong X., Wu Z., Chen Y. (2026). Exploration of Nd-Doped MAPbBr_3_ Single Crystals for Potential Space X-ray Detection. ACS Appl. Opt. Mater..

[29] Chiang Y.-T., Shivarudraiah S.B., Wieczorek A., Khoo K.H., Leong Z., Lim J.W.M., Xing Z., Kumar S., Solari S.F., Li Y.-T. (2025). Understanding Optical Properties and Electronic Structures of High-Entropy Alloyed Perovskite Nanocrystals. Angew. Chem. Int. Ed..

[30] Kung P.-K., Yousuf F., Chen H.-H., Li M.-H., Lin C.-F., Chen P. (2025). Steric Control of Light-Induced Ion Migration Enables Operationally Stable Perovskite Solar Cells. ACS Energy Lett..

[31] Lee J., Park H., Shin S.Y., Lim S.-J., Eun H.J., Jeong B.H., Park S., Lim J., Kim J.H., Lee K.-T. (2025). Synergistic Effect of Compositional Engineering and Graded Heterojunction Formation in Tin-Halide Perovskites for Near-Infrared Photodetectors. Adv. Opt. Mater..

