# Supplementary material for: Synergistic Regulation of Ionic Defects Enabled by NdCl3 for Efficient and Stable Wide-Bandgap Perovskite Solar Cells
Source: Materials (Basel). 2026 Sep 30;19(19):4184. doi: 10.3390/ma19194184 (PMC13665729; doi:10.3390/ma19194184)
Supplement: Supplementary file 1 [file materials-19-04184-s001.zip › materials-4534532-supplementary.pdf]

Supplementary Materials for

Synergistic Regulation of Ionic Defects Enabled by $NdCl_3$ for Efficient and Stable Wide-Bandgap Perovskite Solar Cells

Qi Cui[a#], Haoran Huang[b#], Yu Chen[a], Xu Liu[a], Jincheng Huang[a]*, Jianlin Chen[a], Zhuoyin Peng[a*]

[a] School of Energy and Power Engineering, Changsha University of Science and Technology, Changsha 410111, China

[b] School of Civil and Environmental Engineering, Changsha University of Science and Technology, Changsha 410111, China

[#] The authors contributed equally to this work

*Corresponding authors

E-mail addresses: hjc641@163.com (J. Huang), joeypengzy@outlook.com (Z. Peng)

Figure Section

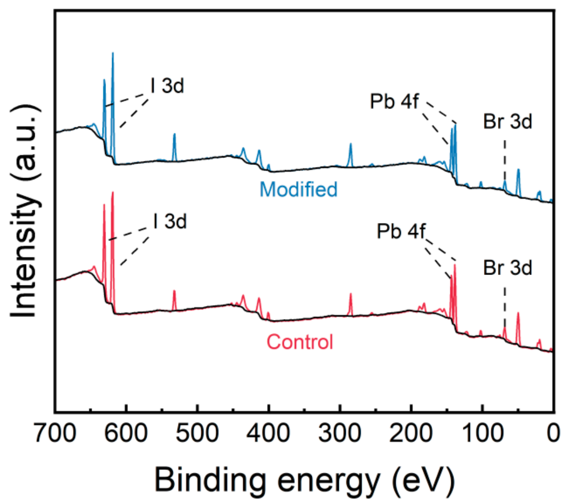

Figure S1. Full XPS survey spectra of control and $NdCl_3$-modified perovskite

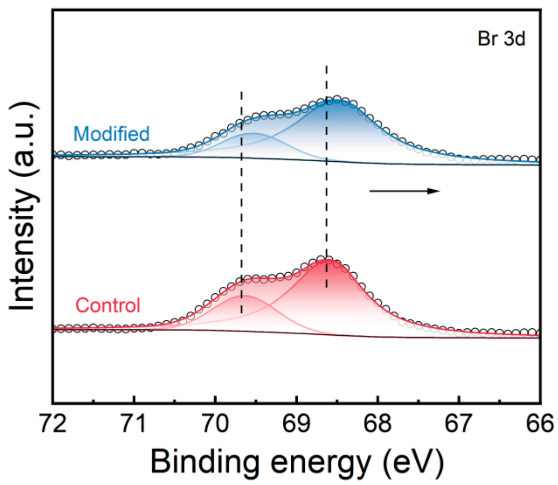

Figure S2. The XPS spectra of Br 3d.

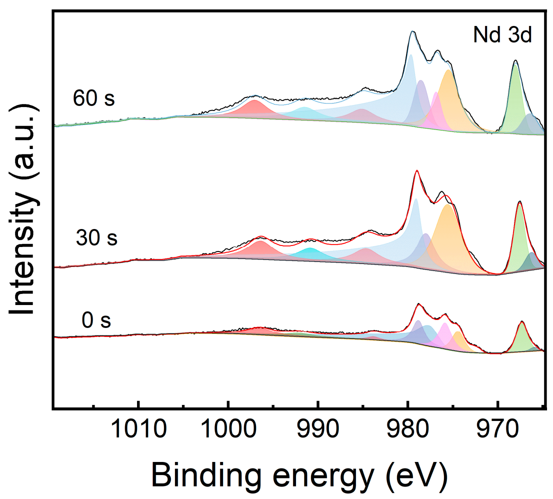

Figure S3. The XPS depth profiling spectra of Nd 3d.

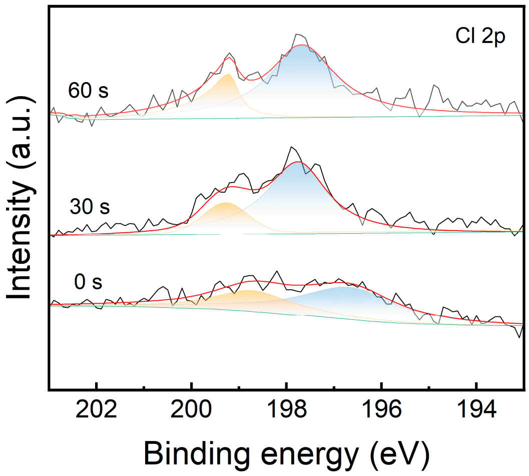

Figure S4. The XPS depth profiling spectra of Cl 2p.

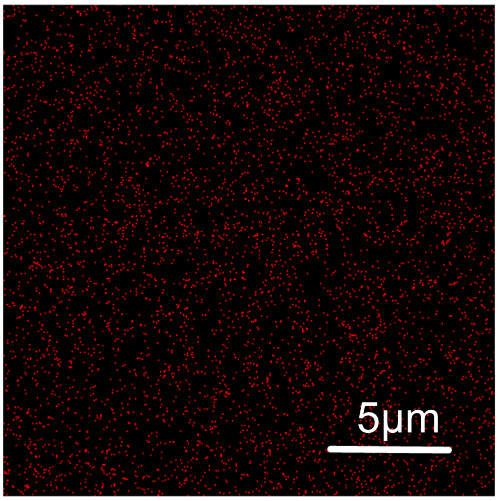

Figure S5. SEM-EDS elemental mapping of Nd.

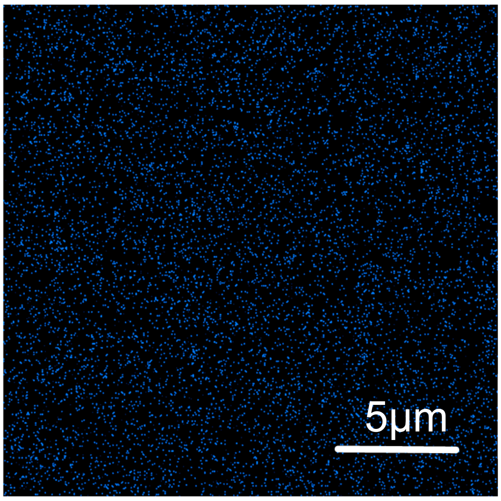

Figure S6. SEM-EDS elemental mapping of Cl.

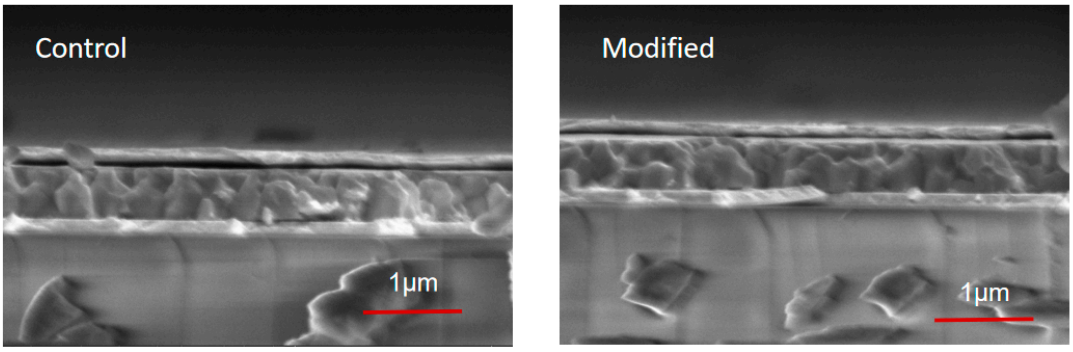

Figure S7. Cross-sectional SEM images.

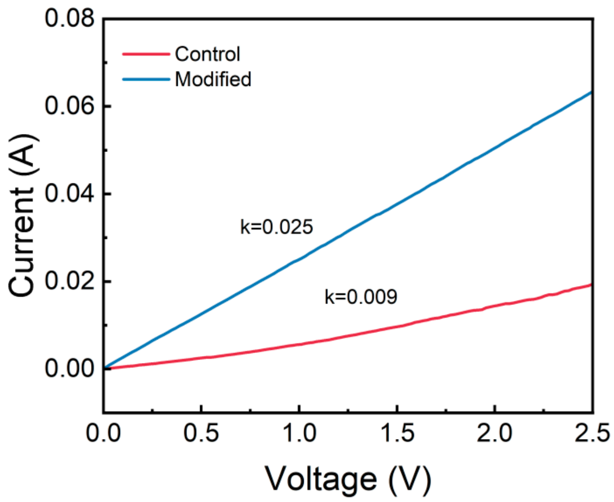

Figure S8. Dark current-voltage (I-V) curves.

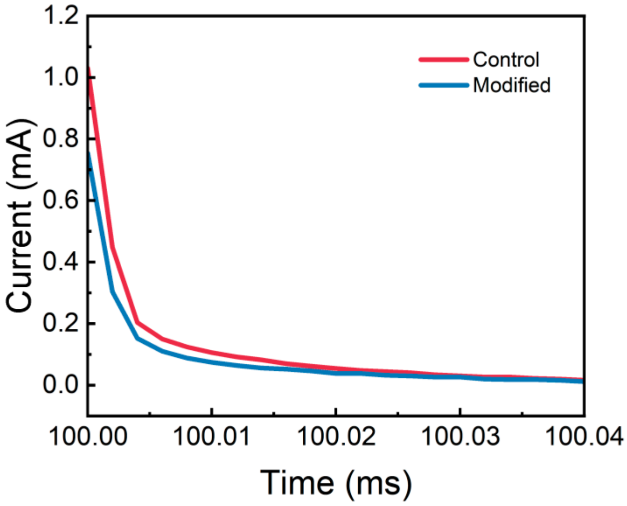

Figure S9. TPC spectra.

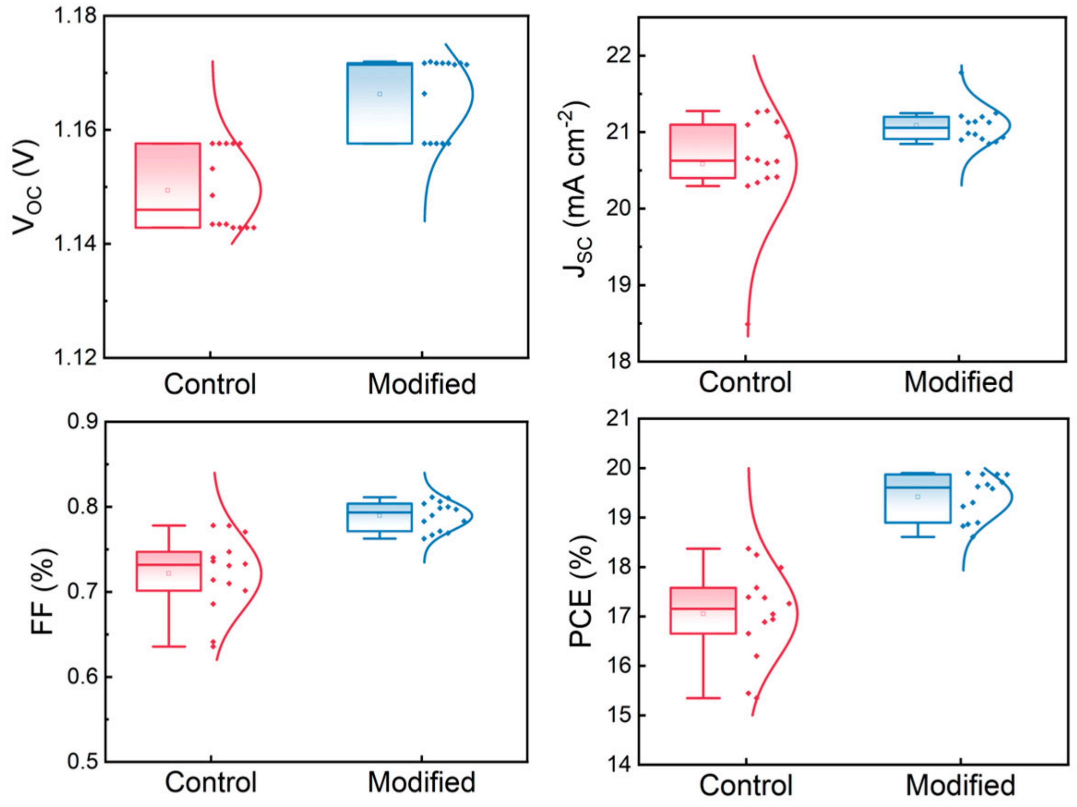

Figure S10. Box plots of V_{OC}, J_{SC}, FF, and PCE for the control device and the modified device.

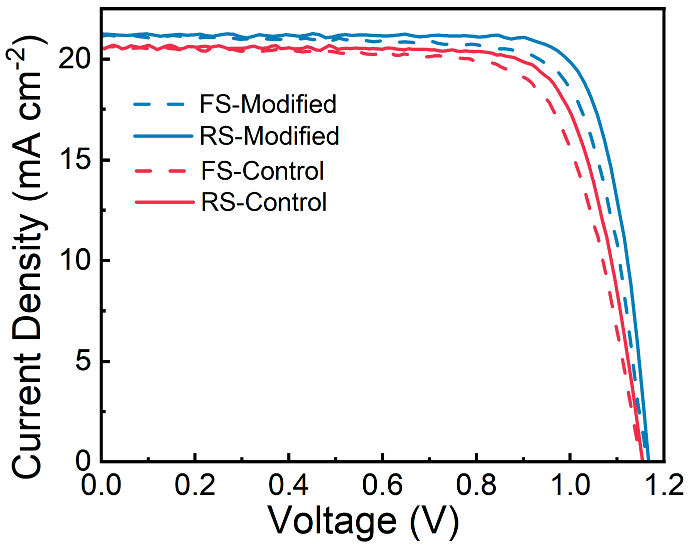

Figure S11. Forward and reverse scan J-V curves

Table S1. XPS spin-orbit splitting energies (Δ) for control and $NdCl_3$-modified perovskite films

Core level	Control Δ (eV)	Modified Δ (eV)
Pb 4f	4.85	4.86
I 3d	11.43	11.42
Br 3d	1.07	1.06

Table S2. EIS fitting parameters for control and $NdCl_3$-modified devices.

Device	R_1 (Ω cm^2)	C_1 (F cm^{-2})	R_2 (Ω cm^2)	C_2 (F cm^{-2})	R_3 (Ω cm^2)	χ^2
Control	1.401	1.686E-007	6.676	0.0001729	0.06417	0.007344
Modified	1.069	1.761E-007	19.33	1.272E-005	1.885	0.001425
